# BRAZILIAN MULTI-SOCIETY POSITION STATEMENT ON EMERGING BARIATRIC AND METABOLIC SURGICAL PROCEDURES

**DOI:** 10.1590/0102-672020230041e1759

**Published:** 2023-09-15

**Authors:** Antonio Carlos Valezi, Antonio Carlos Ligocki Campos, Luiz Carlos Von Bahten

**Affiliations:** 1 Brazilian Society of Bariatric and Metabolic Surgery – São Paulo (SP), Brazil; 2 Brazilian College of Digestive Surgery – São Paulo (SP), Brazil; 3 Brazilian College of Surgeons – Rio de Janeiro (RJ), Brazil.

**Keywords:** Obesity, Bariatric Surgery, Guidelines as Topic, Gastric Bypass, Gastrectomy, Obesidade, Cirurgia Bariátrica, Guias como Assunto, Derivação Gástrica, Gastrectomia

## Abstract

This Brazilian multi-society position statement on emerging bariatric and metabolic surgical procedures was issued by the Brazilian Society of Bariatric and Metabolic Surgery (SBCBM), the Brazilian College of Digestive Surgery (CBCD), and the Brazilian College of Surgeons (CBC). This document is the result of a Brazilian Emerging Surgeries Forum aimed at evaluating the results of surgeries that are not yet listed in the Federal Council of Medicine (CFM), the regulatory agency that oversees and regulates medical practice in Brazil. The Forum integrated more than 400 specialists and academics with extensive knowledge about bariatric and metabolic surgery, representing the three surgical societies: SBCBM, CBC, and CBCD. International speakers participated online and presented their experiences with the techniques under discussion, emphasizing the regulatory policies in their countries. The indications for surgery and the subsequent procedures were carefully reviewed, including one anastomosis gastric bypass (OAGB), single anastomosis duodeno-ileal with sleeve gastrectomy (SADI-S or OADS), sleeve gastrectomy with transit bipartition (SGTB), and sleeve gastrectomy with ileal interposition (SGII). The recommendations of this document are based on an extensive literature review and discussions among bariatric surgery specialists from the three surgical societies. We concluded that patients with a body mass index over 30 kg/m^2^ may be candidates for metabolic surgery in the presence of comorbidities (arterial hypertension and type 2 diabetes) with no response to clinical treatment of obesity or in the control of other associated diseases. Regarding the surgical procedures, we concluded that OAGB, OADS, and SGTB are associated with low morbidity rates, satisfactory weight loss, and resolution of obesity-related comorbidities such as diabetes and arterial hypertension. SGII was considered a good and viable promising surgical alternative technique. The recommendations of this statement aim to synchronize our societies with the sentiments and understandings of most of our members and also serve as a guide for future decisions regarding bariatric surgical procedures in our country and worldwide.

## RESUMO

Esta declaração multissocietária de posicionamento sobre novos procedimentos cirúrgicos bariátricos e metabólicos emergentes foi emitida pela Sociedade Brasileira de Cirurgia Bariátrica e Metabólica (SBCBM), pelo Colégio Brasileiro de Cirurgia Digestiva (CBCD) e pelo Colégio Brasileiro de Cirurgiões (CBC). Este documento é resultado do Fórum Brasileiro de Cirurgias Emergentes, realizado com o objetivo de avaliar os resultados de cirurgias ainda não listadas no Conselho Federal de Medicina (CFM), órgão regulador que fiscaliza e regulamenta a prática médica no Brasil. O Fórum integrou mais de 400 especialistas e acadêmicos com amplo conhecimento sobre cirurgia bariátrica e metabólica, representando as três sociedades cirúrgicas: SBCBM, CBC e CBCD. Palestrantes internacionais participaram online e apresentaram suas experiências com as técnicas em discussão, enfatizando as políticas regulatórias de seus países. As indicações para cirurgia e os procedimentos subsequentes foram cuidadosamente revisados, incluindo *bypass* gástrico de uma anastomose (OAGB), anastomose duodeno-Ileal única com gastrectomia vertical (OADS ou SADI-S), gastrectomia vertical com bipartição de trânsito (SGTB) e gastrectomia vertical com interposição ileal (SGII). As recomendações deste documento são baseadas em extensa revisão da literatura e discussões entre especialistas em cirurgia bariátrica das três sociedades cirúrgicas. Concluímos que pacientes com índice de massa corpórea (IMC) acima de 30 kg/m2 podem ser candidatos à cirurgia metabólica na presença de comorbidades (hipertensão arterial e diabetes tipo 2), sem resposta ao tratamento clínico da obesidade ou no controle de outras doenças associadas. Em relação aos procedimentos cirúrgicos, concluímos que OAGB, OADS e SGTB estão associados a baixas taxas de morbidade e com perda de peso satisfatória e resolução de comorbidades relacionadas à obesidade, como diabetes e hipertensão arterial. A SGII foi considerada uma boa e viável técnica cirúrgica, sendo considerada uma alternativa promissora. As recomendações desta declaração visam sincronizar nossas sociedades com os sentimentos e entendimentos da maioria de nossos membros e também servir como um guia para futuras decisões sobre procedimentos cirúrgicos bariátricos em nosso país e no mundo.

## INTRODUCTION

Obesity is a chronic and progressive disease that has complex and multiple physiological aspects requiring various interventions to advance its treatment and prevention, including bariatric and metabolic surgery (BMS)^6^. The surgical treatment of obesity has solid evidence of efficacy and durability, surpassing other treatment modalities, justifying its substantial increase worldwide.

Similarly to all multifactorial diseases, each obese patient is unique and requires a personalized approach. Individual peculiarities, in different circumstances, lead to indications or restrictions on the use of certain surgical techniques, necessitating a diverse range of procedures to meet the needs and specificities of each patient^10^.

The global rise in revisional surgery can serve as a warning regarding the choices of the primary operation and as a sign of the need for alternatives in the recurrence of the disease after the initial procedure^9,33^. Striving for quality and better outcomes requires a fundamental shift toward value-based care, with a primary focus on the patient and procedures that have demonstrated safety and outcomes that are not inferior to currently regulated procedures^30^.

Our task is to expand the options beyond classical procedures and explore other efficient operations, guided by the wealth of data and physiological knowledge accumulated over years of studying bariatric and metabolic surgery^18^.

As societies dedicated to bariatric procedures and operating in the era of evidence-based medicine, it is undeniable that we have a duty to remain active and keep up with the dynamic evolution of surgical techniques and their outcomes. Brazil plays a significant role in the dissemination of obesity treatments. We must position ourselves regarding the most recent surgical procedures, which have substantial evidence of good practices and satisfactory results for patients.

The Federal Council of Medicine (CFM) has been the regulatory agency overseeing and regulating medical practice in Brazil since 1951^41^. The current CFM resolution (No. 2131/15) exclusively authorizes the following bariatric and metabolic surgeries:

1. Adjustable laparoscopic gastric banding;
2. Vertical gastrectomy;
3. Roux-en-Y gastric bypass; and
4. Biliopancreatic diversion.

We believe that adjustable gastric banding and biliopancreatic diversion (Scopinaro surgery) should be removed from the resolution since they are no longer performed as primary surgical procedures to treat obesity. More recent procedures and even some simple modifications of classic procedures are still considered experimental surgeries by the CFM^11^.

Surgery is an area of constant improvement, from simple tactics to a better understanding of physiological issues, and it is continually evolving. In 2019, the Bariatric Metabolic Surgery Standardization (BMSS) Working Group published an independent consensus about the so-called emerging bariatric surgical procedures, where anatomical aspects and physiological proposals were described, with a wide agreement on their technical settings.

In response to this, in 2022, the International Federation for the Surgery of Obesity and Metabolic Disorders (IFSO) published a position paper on innovative surgical procedures and their ethical implications and results, greatly expanding the list of recommended procedures^23^.

Before that, and with the same rationale, in 2021, the 1st Brazilian Emerging Surgeries Forum (#1BESF) was organized with the objective of updating the statements of our societies, synchronizing them with the sentiments and understandings of the majority of our members, and serving as a guide for future decisions in our country and worldwide.

The #1BESF took place on October 9, 2021, in the capital of São Paulo state, in Brazil. The forum was a collaborative effort between the Brazilian Society of Bariatric and Metabolic Surgery (SBCBM), the Brazilian College of Surgeons (CBC), and the Brazilian College of Digestive Surgery (CBCD). The objective was to examine the bariatric and metabolic surgical procedures used in Brazil and other countries to treat obesity, which are not yet listed in the CFM resolution.

The forum integrated more than 400 specialists and academics with extensive knowledge of bariatric and metabolic surgery, representing the three surgical societies: SBCBM, CBC, and CBCD. International speakers participated online and presented their experiences with the techniques under discussion, emphasizing the regulatory policies in their respective countries.

After evaluating the results of this meeting and considering the current literature evidence, representatives of the sponsoring societies (SBCBM, CBC, and CBCD) realized that it was time to assess certain techniques and express an updated position, which led to the creation of this document. A work subgroup was formed to collect data on safety, reproducibility, and results. Once the work was completed, the following was presented.

### Considerations of regulated, but outdated procedures

CFM resolution No. 1766/2005, amended by No. 1942/2010, still recommends surgical techniques that have fallen into complete disuse in our country with time and results overcome.

In a meta-analysis of long-term follow-up publications, the adjustable gastric band demonstrates a mean excess weight loss of less than 50% (41.75%), failing to meet the current criteria for success in this outcome for the majority of patients^22^. Additionally, the occurrence of moderate to severe complications such as esophageal dilation, band slippage and erosion, gastroesophageal reflux disease, and obesity relapse is not uncommon. As a result, gastric banding has been disregarded as a procedure in our country.

On the other hand, Scopinaro's biliopancreatic diversion shows good rates of weight loss and resolution of metabolic comorbidities but is accompanied by significant nutritional adverse effects. The shortening of the common canal, combined with the potent incretin stimulus, leads to a decrease in the absorption of micronutrients, fat-soluble vitamins, and proteins, and can even cause changes in bowel habits, sometimes with clinical intractability. Due to the imperative need for continuous monitoring and the risk of serious nutritional complications with potentially irreversible consequences, depending on the timing of diagnosis, this procedure has been abandoned^20,44^.

### Beyond body mass index (BMI)

Bariatric procedures remain the most effective and safe intervention for severe obesity. Clinical decision-making should be evidence-based, considering the chronic disease context. A team approach to pre, peri and postoperative care is mandatory, with special attention given to nutritional and metabolic issues.

Various types of metabolic surgery have effectively treated and even prevented type 2 diabetes mellitus (T2DM), reducing the long-term mortality rate when compared to clinical treatment in patients with class III obesity, as shown in large prospective longitudinal studies^9,11,35^.

Class I obesity (body mass index [BMI] between 30 and 35 kg/m^2^) impacts the development of comorbidities, limiting quality of life and reducing longevity. Prospective evidence and large retrospective studies support the possibility of using BMS in patients with class I obesity who have not achieved satisfactory weight loss or adequate control of comorbidities with non-operative treatment^2^.

High-quality data from randomized controlled trials (RCT) establishes that bariatric procedures are more effective than medical or lifestyle interventions in inducing weight loss and initial remission of T2DM, even in patients with initial obesity with a BMI between 30 and 35 kg/m^2 2^.

In 2022, the American Society for Metabolic and Bariatric Surgery (ASMBS) and the IFSO jointly published recommendations on BMS indications. The recommendation regarding the possibility of surgical treatment was extended to patients with a BMI of more than 30 kg/m^2^, and particularly for the Asian population, with a BMI over 27.5 kg/m^2 17^. Importantly, excessive weight loss and generalized malnutrition have not been reported with standard operations in these less obese patients.

The continuous advancement of drug therapy, on the other hand, highlights the need to exhaust clinical treatment attempts in this population and carefully evaluate the severity of the most important comorbidities for clinical outcomes (such as T2DM, arterial hypertension, and their complications) before endorsing a surgical indication^23^.

In updating our statement, we conclude that surgical indication should be based on a high-quality, multidisciplinary decision-making process and should not be limited to individuals with BMI above 35 kg/m^2^. In this regard, we concur with ASMBS and IFSO that patients with a BMI over 30 kg/m^2^ may be candidates for metabolic surgery in the presence of comorbidities (such as arterial hypertension and T2DM) when there is no response to clinical treatment of obesity or control of other associated diseases.

### One anastomosis gastric bypass (OAGB)

**Figure 1:**
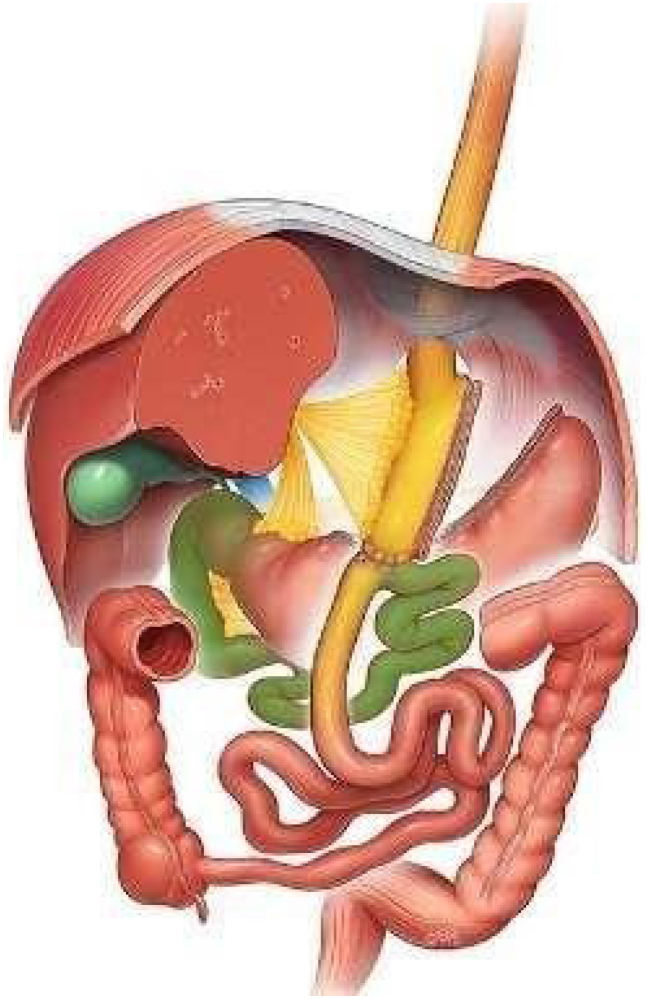
The one-anastomosis gastric bypass.

One anastomosis gastric bypass (OAGB) was first described by Rutledge in 1997 and reported in 2001^23^. The procedure has gained progressive acceptance worldwide with several large series published in the USA, Germany, Italy, Spain, India, Taiwan, Iran, Israel, Egypt, France and the UK (Figure 1).

The IFSO commissioned a task force to determine whether OAGB is a safe and effective procedure as a surgical option for the treatment of obesity and metabolic diseases based on data from 2017, 2018, 2020, and 2021^14–16,26,31^. The UK has a position statement on this procedure that allows surgeons to perform it in their routine practice with scrutiny of their data^32^.

When we evaluate the large volume of publications on OAGB, there is a lack of standardization in its name. It is common to find denominations such as mini gastric bypass, single anastomosis gastric bypass, omega gastric bypass, BAGUA, among others.

During the #1BESF, we concluded that the nomenclature unification is important for scientific and didactic purposes. A multiplicity of names brings confusion and insecurity. Therefore, the name one anastomosis gastric bypass (OAGB) is descriptive of the anatomy and origin and should be preferred. The same principle should be applied to all procedures.

The results of OAGB are quite satisfactory in terms of short surgical time, low perioperative complication rate, weight loss, remission of comorbidities (T2DM, hypertension, sleep apnea, and dyslipidemia), and the outcomes seem at least equivalent to other bariatric surgical procedures^31^. In a comparative, randomized, controlled, multicenter study, 253 patients were assigned between OAGB and (Roux-en-Y) RYGB, which demonstrated non-inferiority of results in weight loss and metabolic improvement^8^. OAGB has been increasingly performed worldwide, as shown by the numerous articles published over the past 20 years; it supports the operation as a fast and effective procedure with excellent results, low complication rates, a shorter learning curve, and total reversibility without significant technical difficulties^8,24,31,36^. Some concerns and controversies are related to the clinical importance of bile reflux and the possibility of significant nutritional deficits related to increased distalization of the common channel compared to traditional RYGB. Despite these considerations, there is no evidence to date of neoplasm incidence related to alkaline reflux in OAGB, although surveillance should be the rule for these patients over time^35^. The low incidence of severe malnutrition and the possibility of common channel alteration in patients with poor nutritional status make evident the procedure's safety^24^.

### Single anastomosis duodeno-ileal bypass with sleeve gastrectomy (SADI-S) or one anastomosis duodenal switch (OADS)

**Figure 2:**
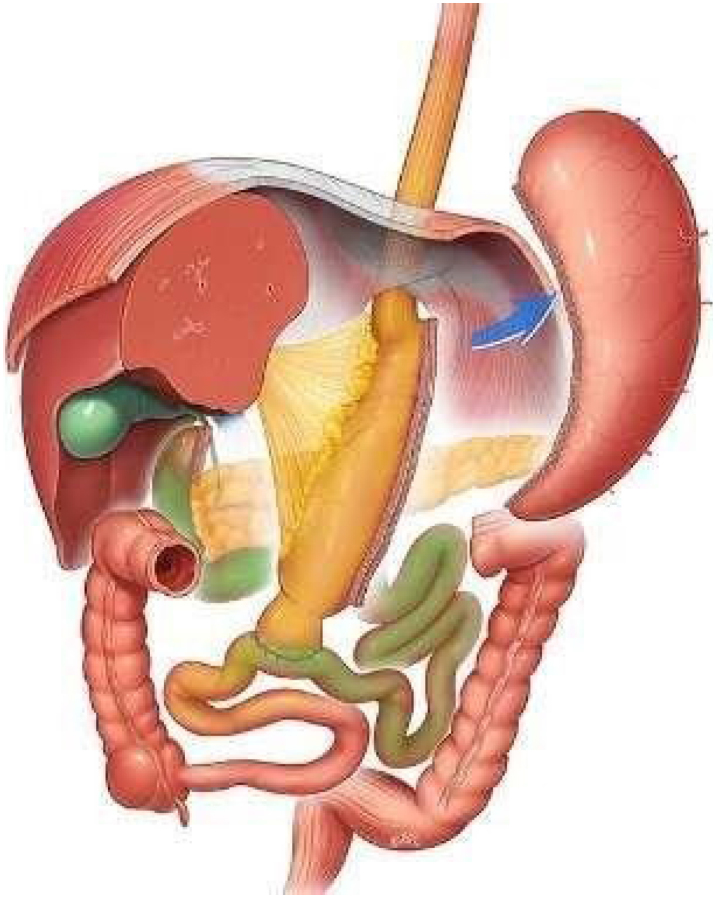
The single anastomosis duodeno-ileal with sleeve gastrectomy or one anastomosis duodenal switch.

Single anastomosis duodeno-ileal with sleeve gastrectomy (SADI-S), also known as one anastomosis duodenal switch (OADS), is a relatively new procedure proposed as a variant of the classic duodenal switch (DS) (Figure 2).

The approach consists of fewer anastomoses, aiming to decrease the overall complexity of the operation and thus the risk of complications^19,29^.

The OADS was proposed by Sánchez-Pernaute et al.^34^ as a modification of DS, anastomosing the duodenum directly to a 250 to 300 cm ileal loop proximal to the ileocecal valve, eliminating the need for the jejunal-ileal anastomosis. The theoretical advantages over DS include reduced operative risk by eliminating one anastomosis, with potentially similar weight loss and benefits with respect to improved comorbidities.

The benefits of OADS as a stand-alone operation for weight loss and long-term diabetic control have become evident^24^. Several authors have used OADS as part of a two-stage procedure along with a vertical gastrectomy in an attempt to increase its effectiveness, especially for super obesity (BMI over 60 kg/m^2^)^40^.

Recently, a systematic review of literature, including 14 studies, reported a mean total body weight loss of 21.5 to 41.2% at 11 months after OADS, with no weight regain after 24 months. The resolution rates for comorbidities were 72.6% for T2DM, 77.2% for dyslipidemia, and 59.0% for hypertension cases. The most common postoperative complication was the reoperation. Despite postoperative nutritional deficiencies in many patients, OADS has proven to be a safe and effective bariatric operation^5,39^. The IFSO conducted a comprehensive literature search and published a solid and compelling article on OADS in 2021^38^.

The task force of #1BESF also evaluated OADS as a tactical possibility for the duodenal switch (DS). Thus, based on the data presented in #1BESF and literature reports, OADS is considered a potential bariatric/metabolic procedure for primary surgery, with particular relevance in cases of revisional surgery due to insufficient weight loss.

### Sleeve gastrectomy with transit bipartition (SGTB)

Sleeve gastrectomy with transit bipartition (SGTB) was described by Santoro in 2002, and the first cases were reported in 2004^37^.

**Figure 3:**
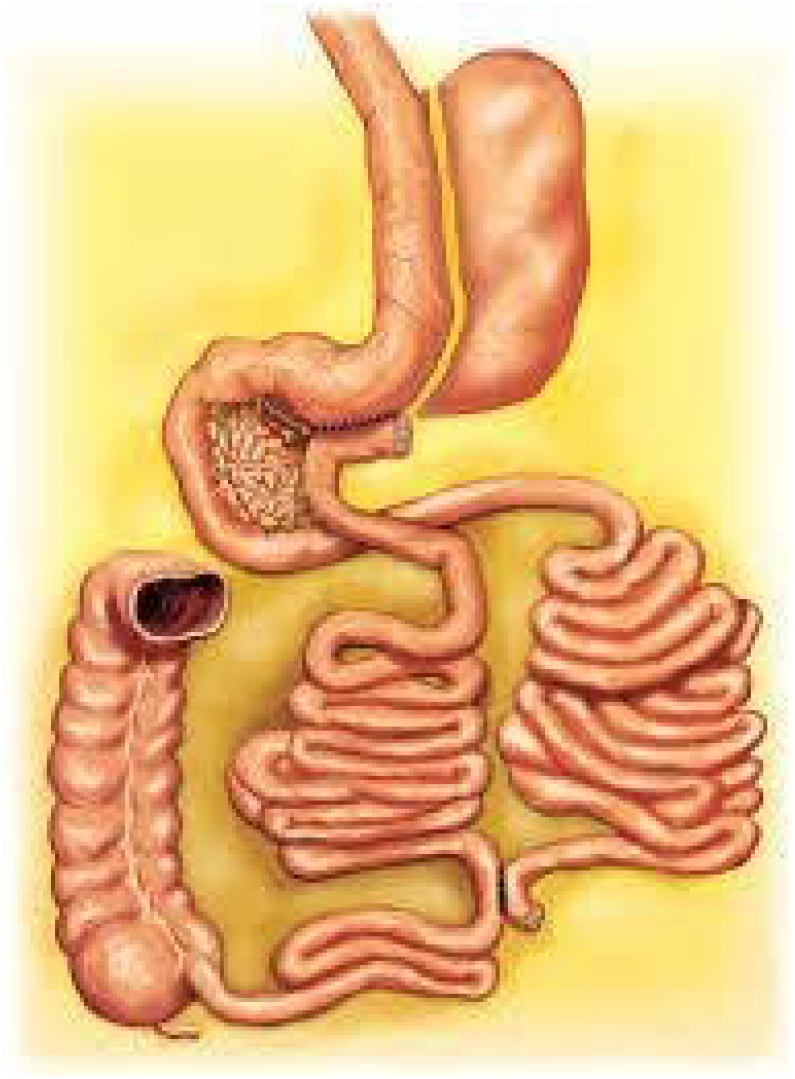
The sleeve gastrectomy with transit bipartition.

This surgical approach creates a gastro-ileal anastomosis in the antrum, allowing partial food drainage from the stomach directly into the ileum. It was designed to spare the duodenum and jejunum from nutrient exclusion (Figure 3).

In 2006, the same authors demonstrated that SGTB could reach significant weight loss without the exclusion of nutrient segments and, therefore, without any blinded endoscopic areas. In fact, it enhanced the postprandial neuroendocrine response. SGTB reduced the production of ghrelin and resistin and allowed for greater absorption of nutrients in the distal portion, resulting in increased secretion of glucagon-like peptide-1 (GLP-1) and peptide YY (PYY). T2DM significantly improved without requiring duodenal exclusion^3,27^.

The procedure has evolved with simplifications. In 2012, Santoro et al. published a significant article presenting five-year results of 1,020 obese patients who underwent SGTB^35^. The results showed important potency, similar to duodenal switch, despite a relatively large gastric pouch with no mechanical obstacles to flow (a well-drained vertical gastrectomy) and no proximal bowel exclusion. The outcomes seem to be primarily achieved through metabolic changes rather than mechanical restriction and malabsorption^19,21^.

SGTB shares some anatomical similarities with DS. However, there is no interruption of duodenal flow, and whereas the goal of DS is malabsorption, the goal of SGTB is to prevent it while maintaining neuroendocrine effects. The option of not performing a duodenal transection is highly favorable in terms of safety and reproducibility. Likewise, not performing intestinal exclusion offers a safer nutritional pattern compared to classical BPD/DS^35^.

SGTB demands fewer steps than DS, because it does not require the duodenum dissection and sectioning, and proximal bowel exclusion. This preserves functions and endoscopic access, and malabsorption is decreased compared to DS^34^.

Azevedo et al.^3^ confirmed, in a randomized controlled trial, the high potency of T2DM remission in SGTB, achieving complete remission in 9 out of 10 patients with severe diabetes, all with BMI below 35 kg/m^2^, and all requiring exogenous insulin. Additionally, they confirmed significant elevations in GLP-1 and PYY, and a significant increase in fibroblast growth factor 19 (FGF-19).

Recently, Topart et al.^43^ compared the results of SGTB and DS and found quite similar weight loss in patients with a BMI equal to or over 50 kg/m^2^, with lower occurrence of diarrhea, shorter surgical time, and better nutrition.

Cagiltay et al.^7^ compared sleeve gastrectomy (SG), OAGB, ileal transposition, and SGTB. They demonstrated that SGTB had the lowest glucose elevations after a mixed meal, with the greatest beta cell responsiveness to glucose measured as ΔC-peptide/Δglucose 0-120 in transit bipartition. SGTB also showed the most significant decrease in glycated hemoglobin. The study highlighted that these findings could be achieved in SGTB with the proximal intestine in transit, which is clearly an advantage.

The concept of bipartition has gained widespread attention, and at least five different surgical tactics are being examined worldwide. They include a transit bipartition (TB) with one anastomosis (OATB), also known as single anastomosis sleeve ileal (SASI), or loop bipartition; the Braun-TB, a TB constructed with the single anastomosis sleeve jejunal (SASJ) instead of the ileum; a bipartition bringing nutrients to the ileum with the anastomosis in the duodenum instead of the antrum; and the isolated TB. These tactics demonstrate the influence and popularity of bipartition concept^28,45^.

Al et al.^1^ published a series of 883 cases with a one-year follow-up in 646 patients, showing no mortality or reoperation, and a complication rate of less than 10.2%, which demonstrates the procedure's safety. SGTB and some of its derived procedures had their standardization published in a World Consensus Meeting Statement in India in 2019^4^.

The #1BESF task force considered the anatomical and physiological aspects of SGTB and verified efficient weight loss and resolution of obesity-related comorbidities based on literature results.

### Sleeve gastrectomy with ileal interposition (SGII)

Over 40 years ago, studies in animal models suggested that the transposition of 10 cm from the terminal ileum to the proximal jejunum would result in weight loss, probably by a mechanism of satiety signaling^14,25^.

**Figure 4:**
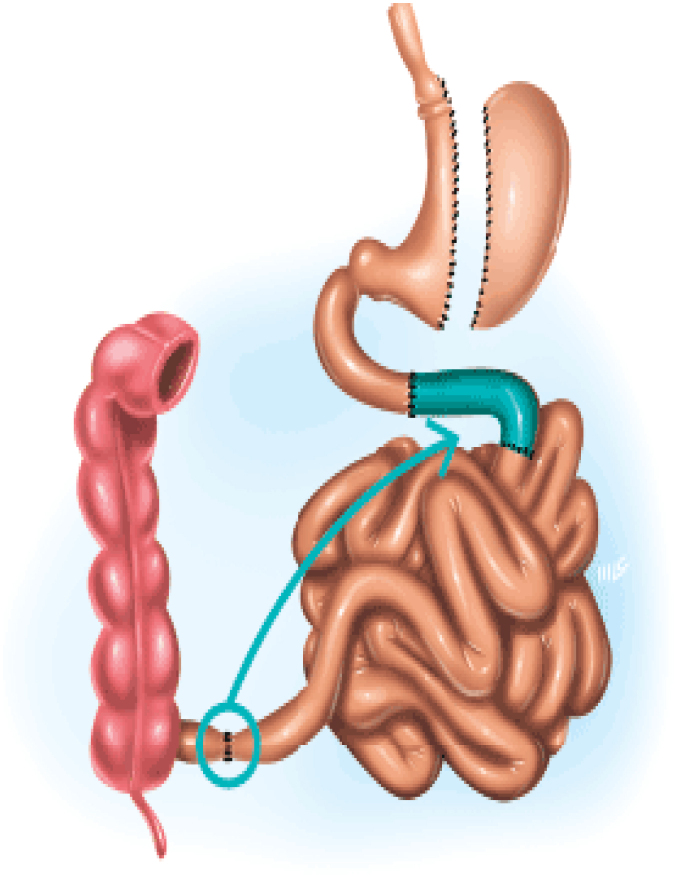
The sleeve gastrectomy with ileal interposition

The proposal of using ileal interposition as a bariatric procedure by DePaula is based on the physiological principle of the ileal brake mechanism, that acts on the neuroendocrine axis of satiety and leads to a sustained reduction in food intake^16,29^ (Figure 4).

In addition to vertical gastrectomy, ileal interposition can be technically performed in the duodenum or proximal jejunum, involving the creation of three anastomoses and, consequently, three mesenteric defects^12,13^.

In an experimental study in animals, the interposition of the ileum demonstrated increased secretion of PYY and GLP-1, improved insulin sensitivity, pancreatic beta cell function, and a positive impact on lipid profile compared to the preoperative period^42^.

DePaula et al.^16^, in a case series of 120 patients published in 2010, reported a mean follow-up of 38 months, showing an 84.5% loss of excess weight, 82.3% resolution of T2DM, and 88.4% resolution of arterial hypertension. Similarly, Tinoco et al.^42^ demonstrated, in a study of 30 patients followed for six to 18 months, an 80% remission rate of T2DM, suggesting interposition as a promising treatment for the disease.

A significant number of publications involving animal models and the physiological basis of the procedure indicate that it is a viable and effective surgical alternative, showing superiority over clinical management in the treatment of obesity and T2DM^31^. However, more publications with higher levels of evidence and longer follow-up are needed to further validate the effectiveness of the ileal interposition procedure.

## CONCLUSIONS

The #1BESF, which took place on October 9, 2021, was a collaborative effort between the SBCBM, the CBC, and the CBCD. The forum concluded that patients with a BMI above 30 kg/m^2^ who have comorbidities should be candidates for metabolic surgery when non-operative obesity treatment or comorbidities control has failed. Concerning surgical procedures, it was concluded that OAGB, OADS, and SGTB are associated with satisfactory weight loss and resolution of obesity-related comorbidities such as T2DM and arterial hypertension. SGII was considered a good and viable promising surgical alternative technique, with results showing superiority over clinical management in the treatment of obesity and T2DM.

The societies that participated in the #1BESF do not have regulatory power, which is exercised by the CFM, advised by its technical chamber. However, as societies, they have the responsibility to inform and guide their members regarding the existing therapeutic possibilities for the treatment of patients with obesity and metabolic disorders. It is clear that physicians, in the exercise of their profession, are free to choose the technique they consider most effective for treating their patients. The promising nature of these treatments necessitates a future reevaluation of this position based on the recommendations of prospective randomized studies comparing them to established techniques, in order to determine their indications for patients.

Finally, surgical procedures emerge and are abandoned as a result of new discoveries, a wider range of therapeutic possibilities, and improved outcomes. Time is the judge of the effectiveness of surgical techniques, and it is not beneficial for science progress to prohibit their use. It is important to emphasize the need for the ongoing collection of accurate data, publication of long-term results, and evaluations from multiple centers and different surgeons, as procedures may emerge while others may fade away over time. It is the duty of surgical societies to periodically come together and reassess their recommendations. Evolution is driven by change.
